# FABP5 is a key player in metabolic modulation and NF-κB dependent inflammation driving pleural mesothelioma

**DOI:** 10.1038/s42003-025-07754-0

**Published:** 2025-02-27

**Authors:** Eleonora Vecchio, Raffaella Gallo, Selena Mimmi, Debora Gentile, Caterina Giordano, Emilio Straface, Rossana Marino, Carmen Caiazza, Arianna Pastore, Maria Rosaria Ruocco, Alessandro Arcucci, Marco Schiavone, Camillo Palmieri, Enrico Iaccino, Mariano Stornaiuolo, Ileana Quinto, Massimo Mallardo, Fernanda Martini, Mauro Tognon, Giuseppe Fiume

**Affiliations:** 1 Department of Experimental and Clinical Medicine, University of Catanzaro “Magna Graecia”, Catanzaro, Italy; 2 Department of Molecular Medicine and Medical Biotechnology, University of Naples “Federico II”, Naples, Italy; 3 Department of Pharmacy, University of Naples Federico II, Naples, Italy; 4 Department of Public Health, University of Naples “Federico II”, Naples, Italy; 5 Department of Molecular and Translational Medicine, Zebrafish Facility, University of Brescia, Brescia, Italy; 6 Department of Medical Sciences, University of Ferrara, Ferrara, Italy

**Keywords:** Cancer metabolism, Mechanisms of disease

## Abstract

Pleural mesothelioma (PM) poses a significant challenge in oncology due to its intricate molecular and metabolic landscape, chronic inflammation, and heightened oxidative stress, which contribute to its notorious resilience and clinical complexities. Despite advancements, the precise mechanisms driving PM carcinogenesis remain elusive, impeding therapeutic progress. Here, we explore the interplay between tumor growth dynamics, lipid metabolism, and NF-κB dysregulation in malignant pleural mesothelioma, shedding light on novel molecular mechanisms underlying its pathogenesis. Our study reveals distinctive growth dynamics in PM cells, characterized by heightened proliferation, altered cell cycle progression, and resistance to apoptosis. Intriguingly, PM cells exhibit increased intracellular accumulation of myristic, palmitic, and stearic acids, suggestive of augmented lipid uptake and altered biosynthesis. Notably, we identify FABP5 as a key player in driving metabolic alterations and inflammation through NF-κB dysregulation in mesothelioma cells, distinguishing them from normal mesothelial cells. Silencing of FABP5 leads to significant alterations in cell dynamics, metabolism, and NF-κB activity, highlighting its potential as a therapeutic target. Our findings unveil a reciprocal relationship between lipid metabolism and inflammation in PM, providing a foundation for targeted therapeutic strategies. Overall, this comprehensive investigation offers insights into the intricate molecular mechanisms driving PM pathogenesis and identifies potential avenues for therapeutic intervention.

## Abstract (web summary)

Pleural mesothelioma (PM) exhibits altered lipid metabolism and NFκB dysregulation, driven by FABP5, promoting tumor growth and inflammation. Silencing FABP5 alters cell dynamics, suggesting it as a potential therapeutic target in PM.

## Introduction

Cells that undergo transformation to a malignant phenotype often have drastic changes in their lipid metabolism. Extensive studies have validated the idea of reprogramming lipid metabolism in cancer^1–3^. Further, tumor cells can enhance de novo lipolysis, fatty acid (FA) uptake, and FA oxidation (FAO) to create energy and accumulate lipids. The main idea behind elevated lipid metabolism in cancer cells is that it is necessary to increase lipids for plasma membrane synthesis and energy production. The mitochondria play a crucial role in the generation of cellular fuel ATP through multiple metabolic processes, including lipid catabolism. Mitochondria are characterized by their high pleomorphism and are considered to be the primary mediators of intracellular energy production^4,5^. Disease progression and drug resistance in cancers can be caused by changes in mitochondrial dynamics^6–8^. In preclinical and early clinical trials, lipid synthesis inhibitors have demonstrated promising anti-cancer effects^9–11^; however, the impact of inhibiting fatty acid synthesis on mitochondrial biology in Pleural mesothelioma (PM) cells is still unclear. PM presents a formidable challenge in oncology, distinguished by its intricate molecular landscape that underscores its notorious resilience and clinical complexities^12–15^. This rare malignancy is hallmarked by chronic inflammation and heightened oxidative stress, yet the mechanisms of its carcinogenesis remain elusive, hampering therapeutic advancements^16–18^. Central to PM’s pathogenesis is a chronic inflammatory state likely due to a strong de-regulation of NF-κB pathway, weaving a complex tapestry of cellular and environmental interactions within a unique tumor microenvironment (TME). Mesothelioma cells, orchestrate the release of macrophage-attractant factors such as interleukin (IL)-8, IL-6, C-C motif chemokine ligand 2 (CCL2/MCP-1), G-CSF, GM-CSF, and MIP-1α, key players in sustaining tumor growth^19^. These factors initiate an inflammatory cascade, recruiting monocytes to the tumor site and shaping the microenvironment conducive to malignant mesothelial cell transformation and proliferation^20^. Chronic oxidative stress emerges as another hallmark of PM carcinogenesis, with reactive oxygen species (ROS) playing a central role in connecting metabolic reprogramming, oxidative stress, and the inflamed microenvironment—critical facets of cancer progression^16–18^. Mitochondria, primarily within tumor cells, generate ROS, influencing various biological processes and signaling cascades, including MAPK, PI3K, and NF-κB^17,18^. Elevated ROS levels can both promote tumor progression and induce macromolecular damage, offering a delicate balance between survival and cell death^16,18,19^. Metabolic plasticity in mesothelioma cells is a noteworthy aspect, with a shift towards aerobic glycolysis regulated by pathways involving MYC, PI3K–AKT, HIF-1α, AMPK, and p53^21,22^. Recent findings also illuminate a selective increase in the translation of mRNAs encoding proteins vital for ribosome assembly and mitochondrial biogenesis, underlining the global impact on mRNA translation, mitochondrial morphology, and metabolic outputs in PM^23^. In the context of these complexities, our study is focused into the in vitro identification of novel molecular mechanisms influencing tumor growth and metabolic parameters, alongside NF-κB activity, revealing a crucial role of the fatty acid transporter FABP5 in driving metabolic alterations and inflammation. In summary, our study explores the interplay between tumor growth dynamics, lipid metabolism, and NF-κB dysregulation in PM, advancing our understanding of PM pathogenesis, and offering prospects for innovative approaches in the treatment of mesothelioma.

## Results

### The cancer growth dynamics in mesothelioma cells links to distinct lipid and mitochondrial metabolic features

Understanding the intricate relationship between tumor growth dynamics and the underlying metabolic properties is imperative for unraveling the complexities of cancer progression. Tumor cells, particularly in malignancies such as mesothelioma, exhibit distinctive growth patterns that can be indicative of underlying metabolic adaptations. Of course, different metabolic phenotypes are a manifestation of tumor plasticity, which is affected by the characteristics of the tumor microenvironment (TME), including nutrient and oxygen availability, pH levels, and interaction with other cells.^24^. Within these adaptations, lipid metabolism reprogramming is a crucial factor in promoting tumorigenesis and cancer progression, as demonstrated by the pronounced alterations in tumors’ lipid signatures^25,26^. Also, oncogenic signaling may interact with dysregulated fatty acid metabolism, which may contribute to tumorigenesis^27^.

Firstly, we asked whether the mesothelioma growth dynamics could correlate with a distinctive profile of specific intracellular free fatty acids. Employing gas chromatography-mass spectrometry (GC-Mass), we conducted an analysis of the intracellular fatty acid content of primary mesothelial HMC35 (Table 1) and compared with that of mesothelioma. We observed an increase in the intracellular content of: (i) specific saturated fatty acids (SFA), including 14:0, 16:0, 18:0, 20:0, and 22:0; (ii) monounsaturated fatty acid (MUFA) 20:1; polyunsaturated fatty acids (PUFA) including 20:2, 16:2; 18:4, and 20:4 in IST-Mes2 and MPP89 compared to normal human primary mesothelial HMC35 (Table 1).

**Table 1:** Intracellular fatty acid composition of primary mesothelial HMC35 and compared with Ist-Mes-2, and MPP89 mesothelioma cell lines from the GC/MS analysis

Lipid Species	Intracellular content of lipid in HMC35 (pg/mg of protein)	Intracellular content of lipid in Ist-Mes-2 (pg/mg of protein)	Intracellular content of lipid in MPP89 cell line (pg/mg of protein)	Statistical significance *p* value *t* Test (HMC vs. Ist-Mes-2)	Statistical significance *p* value *t* Test (HMC vs. MPP89)
SFA (14:0)	3.83 ± 0.47	5.95 ± 0.45	5.56 ± 0.52	*p* < 0.05	*p* < 0.05
SFA (16:0)	80.37 ± 7.22	135.8 ± 6.3	116.85 ± 6.3	*p* < 0.05	*p* < 0.05
SFA (18:0)	71.26 ± 3.20	137.24 ± 8.2	103.48 ± 6.2	*p* < 0.05	*p* < 0.05
MUFA (16:1)	2.43 ± 0.26	3.26 ± 0.45	2.23 ± 0.43	n.s	n.s.
PUFA (18:2)	68.67 ± 5.31	35.52 ± 4.27	50.04 ± 3.84	*p* < 0.05	*p* < 0.05
PUFA (20:4)	25.42 ± 2.22	43.17 ± 6.13	27.39 ± 1.84	*p* < 0.05	n.s.
SFA (12:0)	1.49 ± 0.25	1.15 ± 0.22	1.98 ± 0.28	n.s	n.s.
SFA (20:0)	2.89 ± 0.34	4.39 ± 0.51	3.3 ± 0.37	*p* < 0.05	n.s.
SFA (22:0)	1.95 ± 0.24	3.39 ± 0.33	2.28 ± 0.30	*p* < 0.05	n.s.
MUFA (20:1)	6.2 ± 0.73	8.27 ± 1.24	7.19 ± 1.23	n.s	n.s.
MUFA (22:1)	2.87 ± 0.31	1.89 ± 0.20	1.80 ± 0.41	*p* < 0.05	*p* < 0.05
PUFA (16:2)	1.96 ± 0.23	3.05 ± 0.38	2.78 ± 0.24	*p* < 0.05	*p* < 0.05
PUFA (20:2)	1.53 ± 0.21	3.81 ± 0.32	2.27 ± 0.38	*p* < 0.05	*p* < 0.05
PUFA (22:2)	0.67 ± 0.23	0.87 ± 0.24	0.88 ± 0.19	n.s	n.s
PUFA (18:3)	2.35 ± 0.32	1.16 ± 0.35	2.62 ± 0.34	*p* < 0.05	n.s
PUFA (20:3)	0.94 ± 0.28	1.63 ± 0.34	1.04 ± 0.20	n.s	n.s
PUFA (18:4)	0.55 ± 0.32	2.66 ± 0.41	2.71 ± 0.37	*p* < 0.05	*p* < 0.05
PUFA (20:5)	1.77 ± 0.48	1.6 ± 0.27	1.11 ± 0.32	n.s	n.s
PUFA (22:5)	1.12 ± 0.29	2.07 ± 0.34	1.31 ± 0.26	*p* < 0.05	n.s
PUFA (22:6)	1.42 ± 0.26	0.96 ± 0.39	1.28 ± 0.18	n.s	n.s
Odd (15:0)	1.08 ± 0.25	1.12 ± 0.30	0.91 ± 0.16	n.s	n.s
Odd (17:0)	0.81 ± 0.19	0.99 ± 0.26	0.73 ± 0.11	n.s	n.s
Odd (19:0)	0.34 ± 0.19	0.83 ± 0.34	0.58 ± 0.08	n.s	n.s

Secondly, we proceed to discern a metabolic landscape unbalanced towards increased activity in mesothelioma cells (Ist-Mes-2 and MPP89 cell lines). It is well-known that a hallmark of cancer is an alteration in the metabolic pathways, including glycolysis and the TCA cycle coupled with OXPHOS^28,29^. In this way, adaptation to hypoxia and acidosis conditions via the Warburg phenotype offers an abundant source of precursors for the synthesis of nucleic acids, phospholipids, and fatty acids, which is necessary for tumor cell proliferation^30^. Furthermore, mitochondria are the site of β-oxidation, which turns hydrophobic substrates into useful cellular energy. The fatty acids (FAs) in mitochondria are oxidized as an energy source to produce ATP or heat and provide building blocks for synthesis of biological molecules^31^.

**Fig. 1:**
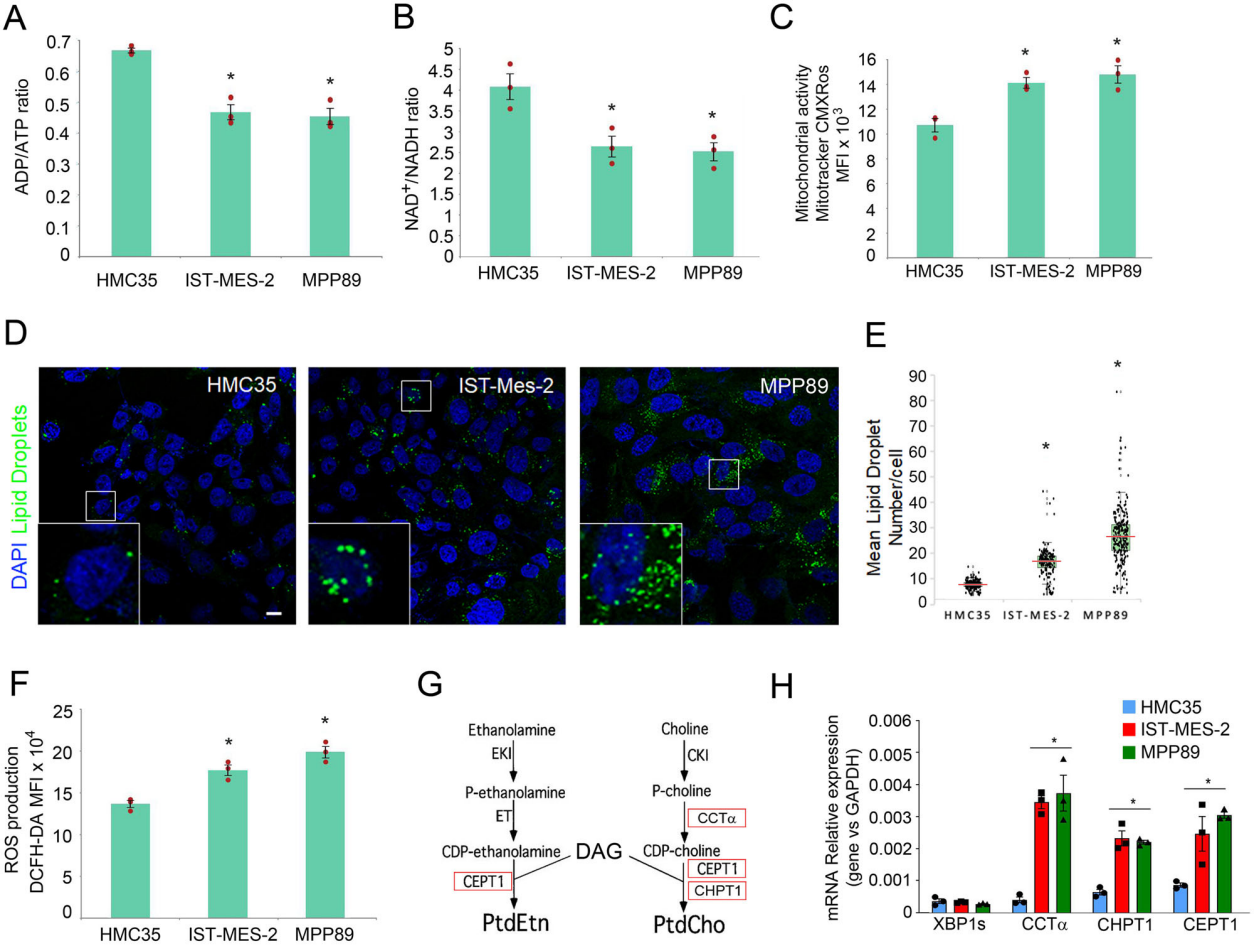
Mesothelioma cell lines showed an impaired metabolic landscape. **A** ADP/ATP Ratio Assay of IST-Mes-2, MPP89, and HMC35 cells was assessed. **B** NAD + /NADH ratio assay of IST-Mes-2, MPP89, and HMC35 cells was shown. **C** A bar diagram showing mitochondrial activity measured by Mitotracker assay. HMC5, IST-Mes-2, or MPP89 cells were stained with mitotracker red for 30 min and then analyzed by flow cytometry. The values were represented by MFI ± SE. **D** Confocal Fluorescence Images showing a higher number of Lipid Droplets per cell in IST-Mes2 and MPP89 compared to HMC35 cells. Images are representative of at least two experiments. Scale bar = 10 μm. **E** Lipid Droplet numbers were normalized to the number of nuclei per analysed image. Statistically significant differences were determined by Student’s *t*-test. Asterisk indicate a value of *p* ≤ 0.01. HMC35 *N* = 223, IST-Mes-2 *N* = 168, MPP89 *N* = 235. **F** A bar diagram showing ROS levels presented as MFI values ± SE. **G** A schematic diagram describes phospholipid biosynthesis pathway. **H** Total RNA of IST-Mes-2, MPP89, and HMC35 cells was analyzed by quantitative real-time PCR for the expression of the key enzymes in phospholipid biosynthesis CCTα, CHPT1, CEPT1. Values were normalized using GAPDH as the housekeeping gene. Data were statistically analyzed by Student’s *t*-test and are reported as mean values ± SE of three independent experiments. The asterisk indicate statistically significant differences between mesothelioma cell lines and primary mesothelial cells.

Based on these notions, we focused our attention on the metabolism reprogramming through the evaluation of broader parameters. To characterize the changes in cellular energy charge (ADP/ATP) and redox (NAD + /NADH) status, the ratios of nucleotides were calculated. The ADP/ATP ratio decreased in IST-Mes2 and MPP89 mesothelioma cells compared to mesothelial HMC35 control cells (Fig. 1A), and NAD+/NADH ratio decreased in IST-Mes2 and MPP89 cells compared to HMC35 cells (Fig. 1B). Moreover, Mitotracker red staining showed a raised mitochondrial mass (Fig. 1C).

Previous reports demonstrated that lipid uptake and storage are also elevated in malignant tumors^31–34^. Since high levels of lipid droplets (LDs) are considered hallmarks of cancer aggressiveness^35^, making cancer cells chemotherapy resistant^36^, we sought to evaluate the lipid droplet formation. BODIPY 493/503 staining revealed an augmented lipid droplet accumulation (Fig.1D, E). As expected, we observed an increase of the reactive oxygen species (ROS) production in mesothelioma cells compared to normal mesothelial cells (Fig.1F). Altogether these data showed an accumulation of lipids within the mesothelioma cells, which impaired cell functionality following mitochondrial dysfunction and increased production of ROS. In cancer cells, the demands of increased membrane biogenesis necessitates a strong up-regulation of lipogenesis^37–40^, being lipids present in large amounts in cellular organelles^41–43^. Furthermore, the higher demand of membrane biogenesis is required to support the secretory pathway, essential for sustaining high protein and lipid trafficking, but also to accommodate the growing cell membrane during rapid cell division. Given that our data suggested lipid accumulation, mitochondrial changes, and increased ROS production, we asked whether the key enzymes involved in phospholipid biosynthesis were upregulated to meet these metabolic demands. Evaluating the expression of the spliced isoform of XBP1, a marker of endoplasmic reticulum stress, and key enzymes in phospholipid biosynthesis (CCTα, CHPT1, and CEPT1)^44^ (Fig. 1G), we discovered a notable increase in mRNA expression of CCTα, CHPT1, and CEPT1 in mesothelioma cell lines without a concurrent alteration in sXBP1 expression (Fig. 1H). Interestingly, the expression of the spliced isoform of XBP1, remained unaltered, suggesting that the observed alterations in metabolic properties were independent of an evident endoplasmic reticulum stress condition. The observed metabolic disparities, including heightened mitochondrial activity and altered phospholipid biosynthesis, underscore the intricate web of metabolic adaptations in mesothelioma, providing a foundation for understanding the disease’s unique metabolic signatures.

### The NF-κB signaling is also impaired in mesothelioma cells

**Fig. 2:**
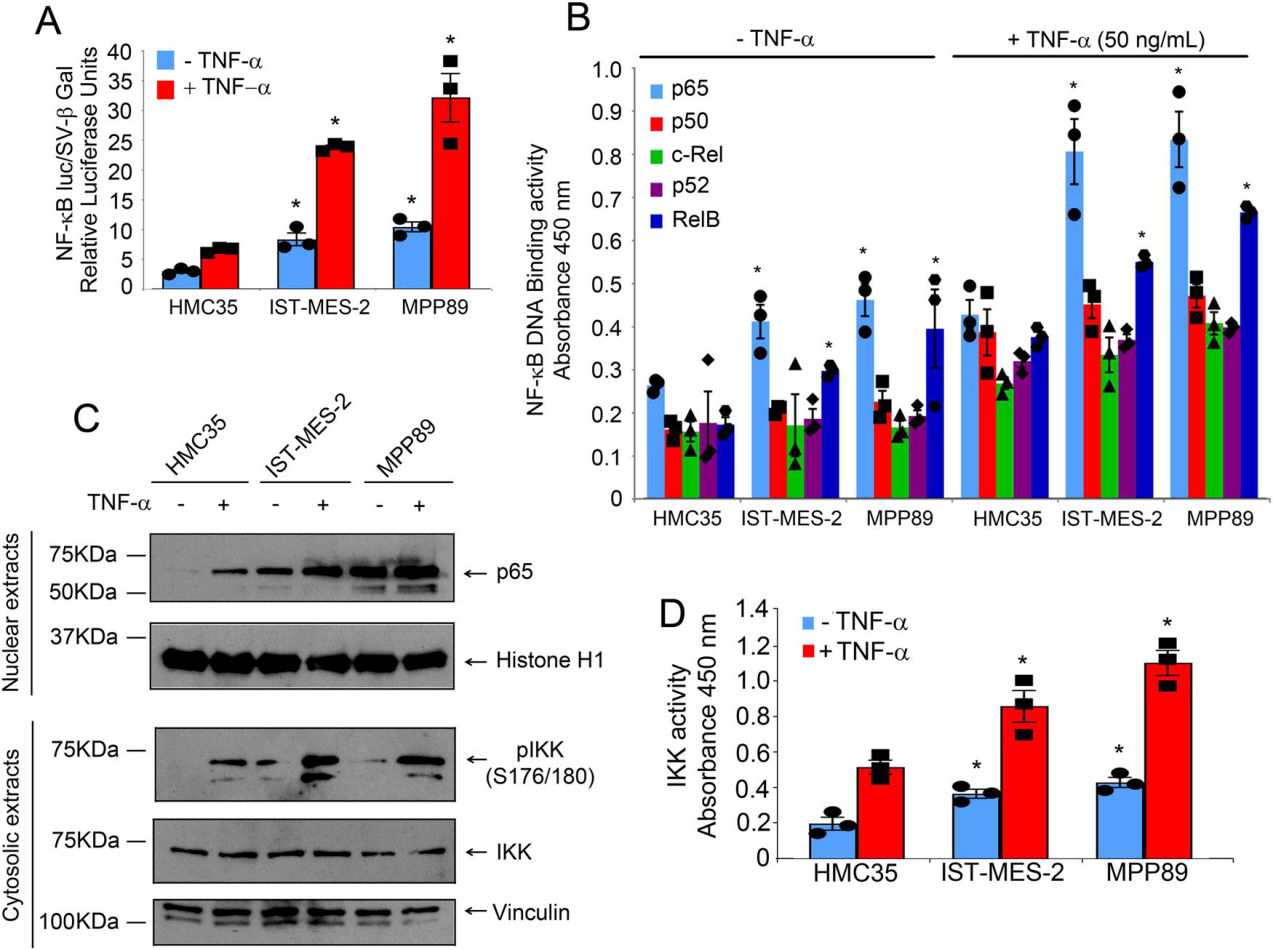
Altered NF-κB activity in mesothelioma cell lines Ist-Mes2 and MPP89 compared to normal mesothelial cells HMC35. **A** IST-Mes-2, MPP89, and HMC35 cells (3 × 10^6^) cells were stimulated or not with TNF-α. After 24 h, cells were co-transfected with pSV-β-Gal (0.2 µg) and pκBluc, (0.2 µg), then were followed for additional 48 h. The ratio of firefly luciferase activity to β-galactosidase activity was expressed as relative light units. Mean values ± SE of 3 independent experiments are shown. **B** The binding of p65, p50, p52, c-Rel, and RelB proteins to the NF-κB double-stranded oligonucleotide was measured in nuclear extracts using the NF-κB Combo Transcription Factor ELISA assay kit. Values are the mean ± SE of three independent experiments. **C** IST-Mes-2, MPP89, and HMC35 cells (3 × 10^6^) cells were stimulated or not with TNF-α for 24 h. p65 was detected by Western blotting of nuclear cell extracts (30 μg). Histone-H1 was included as control of protein loading. Phosphorylated form of IKK and total form of IKK were detected by Western blotting of cytosolic cell extracts (30 μg). Vinculin was included as control of protein loading. **D** Cells were stimulated or not with TNF-α for 24 h and the IKK activity was measured in cytosolic cell extracts (30 μg) using the HTScan IKK kinase assay kit. Values are the mean ± SE of three independent experiments. The asterisks indicate statistically significant differences between stimulated and unstimulated cells, according to Student’s *t*-test (*p* < 0.05).

An established hallmark of mesothelioma lies in its chronic inflammatory nature, and given the intricate relationship between specific metabolic states, notably heightened mitochondrial activity coupled with ROS production, and the NF-κB pathway, we embarked on an investigation into the dysregulation of NF-κB activity in mesothelioma. Employing the NF-κB-Luciferase reporter system, we assessed NF-κB activity in response to TNF-α treatment in both normal mesothelial cells and mesothelioma cell lines. The luciferase activity assay unveiled a robust increase in NF-κB activity in IST-Mes2 and MPP89 compared to HMC35. Interestingly, the presence of TNF-alpha exacerbated this increase, indicating a heightened responsiveness in mesothelioma cells (Fig. 2A). Next, we employed an ELISA EMSA test to assess the DNA-binding activity of each NF-κB subunits, in the different cell lines, revealing a notable activity of DNA binding of p65 and RelB, which was intensified upon TNF-α treatment in mesothelioma cell lines IST-Mes2 and MPP89 (Fig. 2B). Next, we explored the molecular mechanisms behind NF-κB activation, by monitoring nuclear translocation of p65 and the phosphorylation status of IKK at serine 176/180. Mesothelioma cell lines exhibited an augmented nuclear translocation of p65 and increased phosphorylation of cytosolic IKK, which were exacerbated upon TNF-alpha treatment. Importantly, the levels of expression of cytosolic IKK remained unaltered (Fig. 2C). The IKK Kinase Assay corroborated these findings, demonstrating increased IKKalpha/beta activity in mesothelioma cell lines compared to normal mesothelial cells, both in the absence and presence of TNF-alpha (Fig. 2D).

### FABP5 promotes the expression levels of inflammatory genes via activation of the nuclear factor-kappa B (NF-κB) signaling pathway

**Fig. 3:**
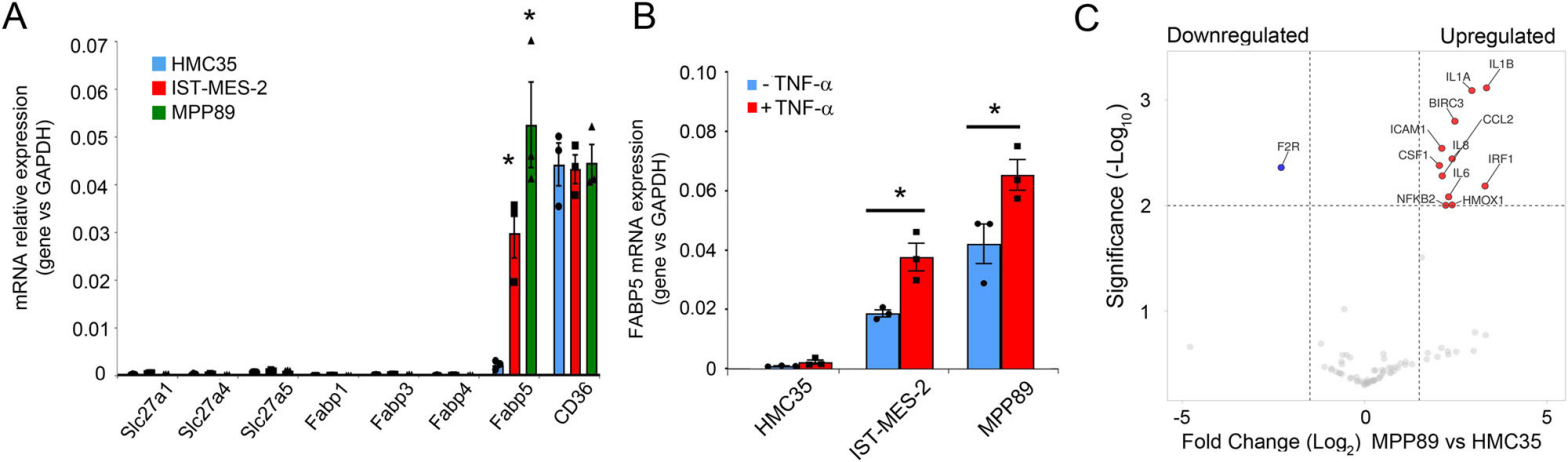
The expression of FABP5 increases in mesothelioma cell lines. **A** Total RNA of IST-Mes-2, MPP89, and HMC35 cells was analyzed by quantitative real-time PCR for the expression of the key fatty acid transporters Slc27a1, Slc27a4, Slc27a5, Fabp1, Fabp3, Fabp4, Fabp5, and CD36. Values were normalized using GAPDH as the housekeeping gene. Data were statistically analyzed by Student’s *t*-test and are reported as mean values ± SE of three independent experiments. **B** Total RNA of IST-Mes-2, MPP89, and HMC35 cells stimulated or not with TNF-α for 24 h was analyzed by quantitative real-time PCR for the expression of FABP5. Values were normalized using GAPDH as the housekeeping gene. Data were statistically analyzed by Student’s *t*-test and are reported as mean values ± SE of three independent experiments. The asterisk indicates statistically significant differences between mesothelioma cell lines and primary mesothelial cells. **C** Volcano plot indicates the gene expression profile of MPP89 mesothelioma compared to normal mesothelial HMC35 cells. Lower expressed genes were indicated with blue, while higher expressed genes were indicated with red.

Within the complex word of lipid metabolism, our exploration expanded beyond the intracellular and extracellular content of free fatty acids to investigate the potential dynamics of fatty acid uptake in mesothelioma cell lines compared to their normal mesothelial counterparts. To scrutinize this, we probed the expression of key fatty acid transporters Slc27a1, Slc27a4, Slc27a5, Fabp1, Fabp3, Fabp4, Fabp5, and CD36 (Fig. 3A). Intriguingly, CD36 emerged as the predominantly expressed fatty acid transporter in both normal and mesothelioma cell lines. However, a notable distinction unfolded with the increased and specific expression of FABP5 in mesothelioma cell lines, presenting a potential contributor to the observed increase in free fatty acid uptake.

Previous papers^45–47^ showed that FABP5 could promote the activation of NF-κB activity in prostate and breast cancer cell lines and FABP5 deficiency could impair macrophages mediated inflammation. We questioned ourselves: could the expression of FABP5 be linked with NF-κB activity in mesothelioma cells?

The mRNA expression levels of FABP5, measured in the presence or absence of TNF-alpha treatment, revealed a correlation with NF-κB activity, emphasizing the potential role of FABP5 in driving NF-κB dysregulation (Fig. 3B). The downstream impact of NF-κB dysregulation was explored through the analysis of 90 inflammatory NF-κB dependent genes. Notably, in mesothelioma cell line MPP89 compared to normal mesothelial cells HMC35, we observed a substantial increase in the expression of key inflammatory genes, including IL1A, IL1B, IL6, IL8, CCL2, ICAM, NFKB2, HMOX, BIRC3, CSF, and IRF1, compared to normal primary mesothelial cells HMC35 (Fig. 3C). Taken together, our data show the multifaceted dysregulation of the NF-κB pathway in mesothelioma, shedding light on activation mechanisms and downstream inflammatory signatures, with potential implications for understanding and targeting the inflammatory milieu in this malignancy.

### FABP5 silencing affects mesothelioma cell dynamics, metabolism, and NF-κB activity

**Fig. 4:**
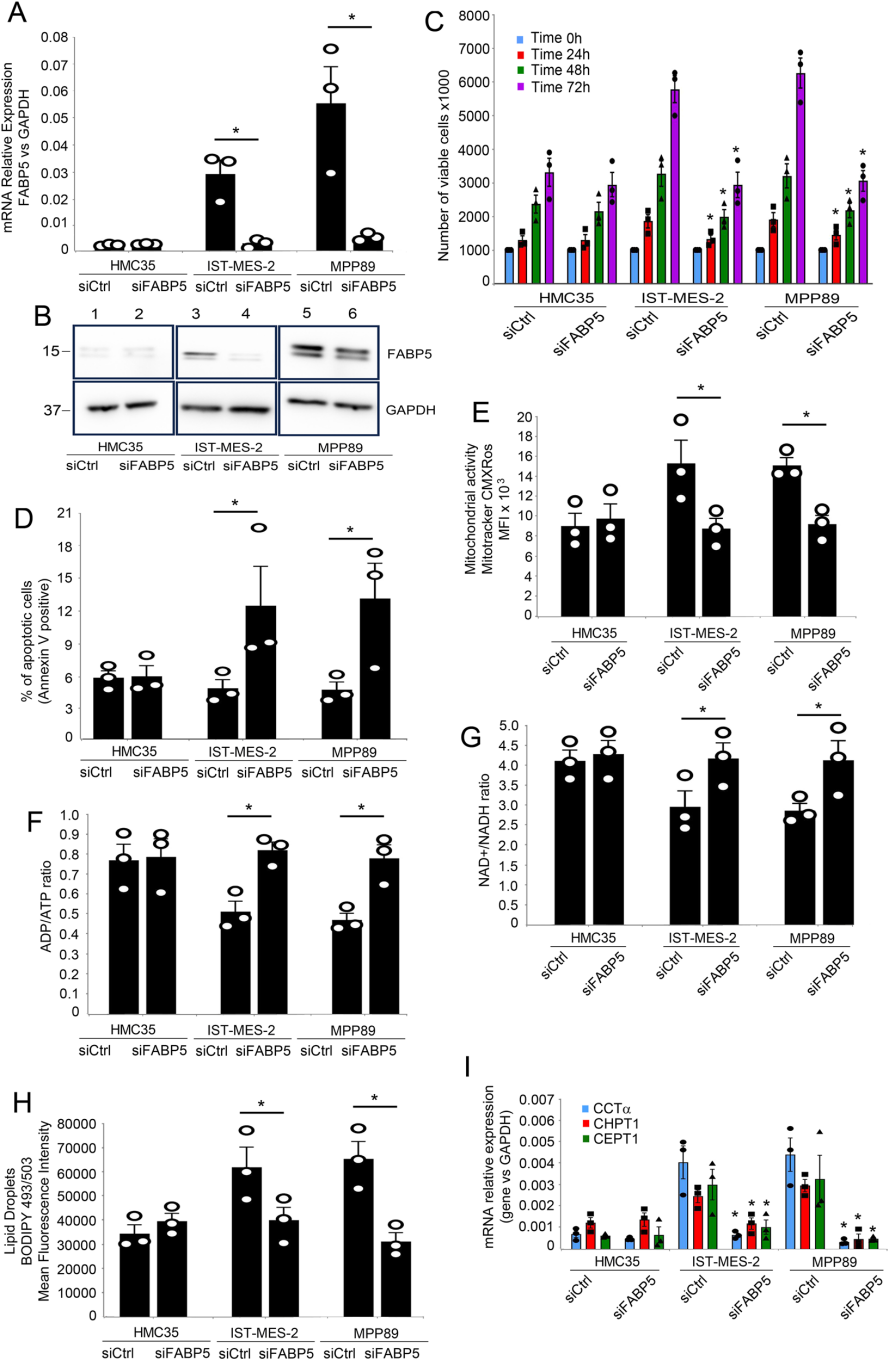
FABP5 depletion affects Mesothelioma Cell Dynamics and Metabolism. **A** Total RNA of IST-Mes-2, MPP89, and HMC35 cells were transfected with siRNA FABP5 or scrambled siRNA (200 pmoles) and analyzed by quantitative real-time PCR for the expression of FABP5. Values were normalized using GAPDH as the housekeeping gene. Data were statistically analyzed by Student’s *t*-test and are reported as mean values ± SE of three independent experiments. The asterisks indicate statistically significant differences between cells transfected with siRNA FABP5 or scrambled siRNA, according to Student’s *t*-test (*p* < 0.05). **B** Western blotting analysis of HMC35, IST-Mes-2, and MPP89, transfected with siRNA FABP5 or scrambled siRNA (200 pmoles), whole cell extracts (50 µg) by using FABP5 or GAPDH antibodies. **C** Cell proliferation of IST-Mes-2, MPP89, and HMC35 cells transfected with siRNA FABP5 or scrambled siRNA (200 pmoles) was measured after 24, 48, and 72 h by Trypan Blue dye exclusion. **D** Apoptotic cells (Annexin V positive cells) were assessed by Annexin V binding assay. Values (mean ± SE; *n* = 3) are shown. Statistically significant difference was determined by Student’s *t*-test and was indicated by the asterisks (*p* < 0.05). **E** A bar diagram showing mitochondrial activity measured by Mitotracker assay. HMC5, IST-Mes-2, or MPP89 cells transfected with siRNA FABP5 or scrambled siRNA were stained with mitotracker red for 30 min and then analyzed by flow cytometry. The values were represented by MFI ± SE. **F** ADP/ATP Ratio Assay of IST-Mes-2, MPP89, and HMC35 cells transfected with siRNA FABP5 or scrambled siRNA was assessed. **G** NAD+/NADH ratio assay of IST-Mes-2, MPP89, and HMC35 cells transfected with siRNA FABP5 or scrambled siRNA (200 pmoles) was shown. **H** A bar diagram showing lipid droplets was assessed by Bodipy assay and represented as MFI values ± SE. Values (mean ± SE; *n* = 3) are shown. Statistically significant difference was determined by Student’s *t*-test. **I** Total RNA of IST-Mes-2, MPP89, and HMC35 cells transfected with siRNA FABP5 or scrambled siRNA (200 pmoles) was analyzed by quantitative real-time PCR for the expression of the key enzymes in phospholipid biosynthesis CCTα, CHPT1, CEPT1. Values were normalized using GAPDH as the housekeeping gene. Data were statistically analyzed by Student’s *t*-test and are reported as mean values ± SE of three independent experiments. The asterisk indicates statistically significant differences between mesothelioma cell lines and primary mesothelial cells.

**Fig. 5:**
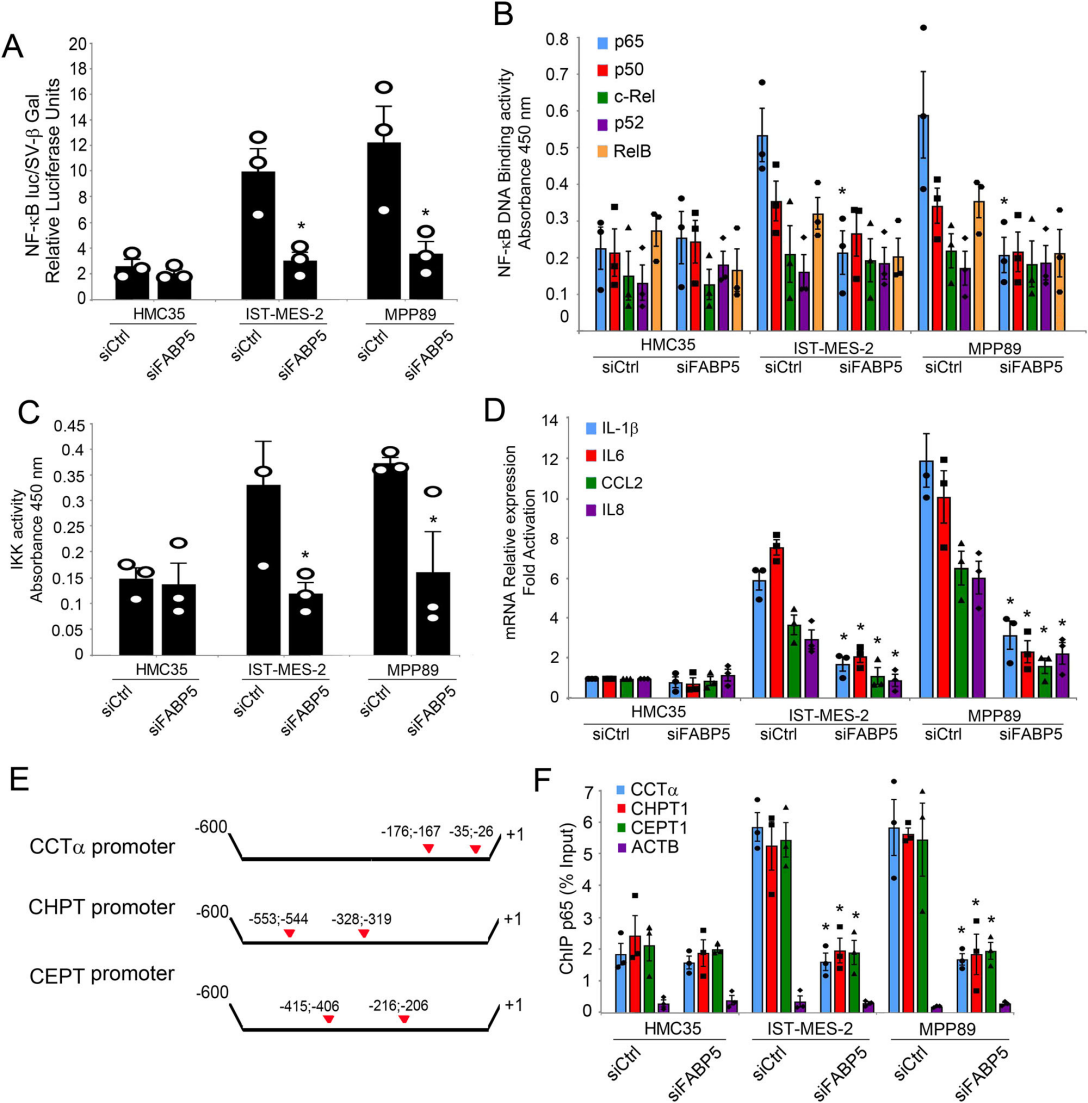
FABP5 depletion affects NF-κB activity. **A** HMC35, IST-Mes-2, and MPP89 cells (3 × 10^6^) were transfected with siRNA FABP5 or scrambled siRNA (200 pmoles) in presence of pSV-β-Gal (0.2 mg) and pκBluc, (0.2 mg). Forty-eight hours later, firefly luciferase activity and β-galactosidase activity were measured. The ratio of firefly luciferase activity to β-galactosidase activity was expressed as relative light units. Mean values ± SE of 3 independent experiments are shown. **B** HMC35, IST-Mes-2, and MPP89 cells (3 × 10^6^) were transfected with siRNA FABP5 or scrambled siRNA (200 pmoles). The binding of p65, p50, p52, c-Rel, and RelB proteins to the NF-κB double-stranded oligonucleotide was measured in nuclear extracts using the NF-kB Combo Transcription Factor ELISA assay kit. Values are the mean ± SE of three independent experiments. **C** HMC35, IST-Mes-2, and MPP89 cells (3 × 10^6^) were transfected with siRNA FABP5 or scrambled siRNA. IKK activity was measured in cytosolic cell extracts (30 μg) using the HTScan IKK kinase assay kit. Values are the mean ± SE of three independent experiments. The asterisks indicate statistically significant differences between stimulated and unstimulated cells, according to Student’s *t-*test (*p* < 0.05). **D** HMC35, IST-Mes-2, and MPP89 cells (3 × 10^6^) were transfected with siRNA FABP5 or scrambled siRNA (200 pmoles). Total RNA of IST-Mes-2, MPP89, and HMC35 cells was analyzed by quantitative real-time PCR for the expression of NF-κB dependent genes *IL-1β*, *IL6*, *CCL2,* and *IL8*. **E** Schematic representation of CCTα, CHPT1, and CEPT1 promoters. Red arrows indicate the NF-κB enhancers with the related positions. **F** HMC35, IST-Mes-2, and MPP89 cells (3 x 10^6^) were transfected with siRNA FABP5 or scrambled siRNA (200 pmoles). 48 h later, ChIP was performed with anti-p65 antibody. Real-time PCR was performed with primers specific for the indicated promoters. Values (mean ± SE, *n*  =  3) are shown. The asterisks indicate statistically significant differences compared to the control (siControl) according to the Student’s *t-*test (*p* <  0.05).

Given the pivotal role attributed to FABP5 and envisioning its potential as a therapeutic target in the context of innovative strategies against mesothelioma, we investigated the consequences of FABP5 silencing on the proliferation, apoptosis, and metabolism of both mesothelial and mesothelioma cells. Inducing FABP5 silencing, using a pool of siRNA against FABP5 or a scrambled siRNA, as control (Fig. 4A), we observed a notable reduction in the proliferation rates of IstMes2 and MPP89, while the proliferation of normal mesothelial cells HMC35 remained unaffected (Fig. 4B). Remarkably, FABP5 silencing induced a significant increase in apoptosis, as evidenced by Annexin-V assay, in both mesothelioma cell lines IstMes2 and MPP89, while apoptosis levels in normal mesothelial cells remained unaltered (Fig. 4C and Supplementary Fig. 2A, B). Further, we found FABP5 silencing triggered notable metabolic shifts in mesothelioma cells. Specifically, we observed a decrease in mitochondrial activity (Fig. 4D), accompanied by an increase in the ADP/ATP ratio (Fig. 4E) and the NAD/NADH ratio (Fig. 4F). In contrast, normal mesothelial cells exhibited no discernible changes in these metabolic parameters. Furthermore, FABP5 silencing led to a reduction in the abundance of lipid droplets (Fig. 4G and Supplementary Fig. 3C, D) and a concurrent decrease in the mRNA expression levels of key enzymes involved in phospholipid biosynthesis CCTα, CHPT1, and CEPT1 (Fig. 4H). These findings underscore the potential therapeutic efficacy of targeting FABP5, revealing its intricate involvement in mesothelioma cell dynamics and metabolic pathways. The observed alterations in proliferation, apoptosis, and metabolism upon FABP5 silencing highlight its significance as a prospective therapeutic target in the pursuit of novel strategies against mesothelioma. Building on our investigation into the consequences of FABP5 silencing on mesothelioma cell dynamics and metabolism and on previous reports indicating that FABP5 induces an increased FAs synthesis leading to NF-κB activation via production of ROS derived from β-oxidation in mitochondria^48–50^, we delved into its influence on NF-κB activity and downstream targets. Utilizing the NF-κB-Luciferase reporter system, we transfected mesothelioma cell lines with FABP5 siRNA or control scrambled siRNA. Strikingly, FABP5 silencing led to a significant reduction in NF-κB activity in mesothelioma cell lines, aligning the levels with those observed in normal mesothelial cells (Fig. 5A). Employing ELISA EMSA, we delineated the impact of FABP5 silencing on the DNA binding activity of NF-κB subunits. Notably, FABP5 silencing selectively reduced the DNA binding activity of p65, without affecting p50, cRel, and RelB (Fig. 5B). Additionally, FABP5 silencing resulted in a decrease in IKK activity in mesothelioma cell lines (Fig. 5C). Based on our earlier identification of upregulated NF-κB dependent cytokines in mesothelioma, we explored the effect of FABP5 silencing on their expression. FABP5 silencing led to the downregulation of NF-κB dependent genes IL1B, IL6, CCL2, and IL8 (Fig. 5D), suggesting a potential anti-inflammatory impact. Given the elevated mRNA expression of phospholipid biosynthesis genes (CCTα, CHPT1, and CEPT1) in mesothelioma cell lines, we investigated the putative regulatory role of NF-κB. Computational analysis using JASPAR identified NF-κB enhancers in the promoters of these genes. We found two NF-κB enhancers in CCTα (−35/−26; −176/−167 compared to transcriptional start site), in CHPT1 (−553/−544; −328/−319), and in CEPT1 (−406/−415; −216/−206) promoters (Fig. 5E). Subsequently, ChIP assays revealed increased recruitment of p65 to the NF-κB enhancers of CCTα, CHPT1, and CEPT1 in mesothelioma cell lines compared to normal mesothelial cells (HMC35) (Fig. 5F). Crucially, FABP5 silencing attenuated this recruitment, suggesting a nuanced modulation of NF-κB activity by FABP5 (Fig. 5F). These findings underscore the intricate interplay between FABP5, NF-κB activity, and downstream targets, providing valuable insights into the potential mechanisms underlying the therapeutic impact of FABP5 modulation in mesothelioma.

## Discussion

The profound alterations in cellular metabolism, a hallmark of cancer, play a pivotal role in the initiation, progression, and sustained growth of malignancies. Among the myriad metabolic changes observed in cancer cells, dysregulation in lipid metabolism stands out as a crucial contributor to the complex cellular reprogramming that characterizes tumorigenesis. Understanding the importance of metabolic alterations, especially those related to lipid metabolism, is essential for unraveling the intricate mechanisms driving cancer progression^51–54^. Our investigation into the interplay of growth dynamics, lipid metabolism, and NF-κB dysregulation in mesothelioma sheds light on the intricate mechanisms contributing to the distinctive characteristics of this malignancy. The observed alterations in fatty acid synthesis correlated to cancer growth highlight the unique growth dynamics of mesothelioma cells compared to normal mesothelial cells. This increased intracellular accumulation not only signifies an enhanced uptake but could also be indicative of elevated biosynthesis and altered intracellular mobilization of these lipids^55–57^. Such alterations may contribute to intensified phospholipid biosynthesis, supporting essential cellular processes such as cell proliferation and the secretory pathway^55–57^. Particularly noteworthy is the potential role in facilitating the secretion of inflammatory cytokines, thereby establishing a link between lipid metabolism and the inflammatory milieu associated with mesothelioma. This altered lipidomic profile may contribute to the robust growth dynamics observed in mesothelioma, implicating a potential link between lipid metabolism and tumorigenesis^55–57^. The identified overexpression of the fatty acid transporter FABP5 (Fig. 3A) in mesothelioma cells further supports this association, as FABP5 is known to play a crucial role in fatty acid uptake^58,59^ and metabolism in various cancers. More specifically, FABP5 has been shown facilitate the uptake of exogenous fatty acids and de novo lipid synthesis, thus promoting mitochondrial FAO and membrane lipid synthesis, respectively^60^. Moreover, FABP5 deficiency is associated with enhanced immune suppressive activity of Tregs and with a reduction of inflammatory status^60^. In normal physiology, FABP5 is known to function as a cytosolic transporter for oleic acid (OA)^58,59^ and it has been reported that exposure to OA leads to an upregulation of FABP5 expression in both normal prostate cells and pancreatic islet cells^47^. In addition to the role of FABP5 in metabolic diseases, FABP5 may also play a critical role in cancer progression. FABP5 is overexpressed in several types of cancer and associated with poor prognosis^61–66^. Recently, it has been shown that in hepatocellular carcinoma (HCC) tissues, FABP5 induced HIF-1α expression and activity, and their expression levels were associated with poor prognosis^47^. The metabolic characterization of mesothelioma cells extends beyond lipid metabolism to encompass mitochondrial mass (Fig. 3B), ADP/ATP (Fig. 3C), and NAD+/NADH (Fig. 3D) ratios, ROS production (Fig. 3E), lipid droplet accumulation (Fig. 3F). The heightened mitochondrial activity observed in mesothelioma cells aligns with the Warburg effect, a phenomenon commonly observed in cancer cells wherein energy production shifts towards glycolysis, even in the presence of oxygen^67^. Consistent with previous reports^68^, our observations about the altered ratios of ADP/ATP and NAD+/NADH signify disruptions in energy homeostasis, potentially contributing to the sustained growth and viability of mesothelioma cells. The increased ROS production and lipid droplet accumulation provide additional facets of the metabolic adaptations in mesothelioma, indicating potential avenues for therapeutic interventions. It is notable that cytosolic fat is stored in lipid droplets, which play crucial roles in energy production, ROS protection, and membrane biogenesis essential for facilitating rapid cancer cell proliferation. Accumulation of lipid droplets has been documented across a spectrum of cancers, encompassing breast, brain, bile-duct, cervical, colon, liver, lung, ovarian, prostate, and pancreatic cancer, as well as clear-cell renal cell carcinoma^47^.

Our study delves into the intricate dysregulation of the NF-κB pathway in mesothelioma, unraveling a cascade of events that contribute to the pro-inflammatory milieu characteristic of this malignancy. The heightened NF-κB activity, observed even in the absence of TNF-α treatment, suggests intrinsic dysregulation within mesothelioma cells (Fig. 4). The specific activation of NF-κB subunits p65 and RelB, coupled with increased nuclear translocation and IKK phosphorylation, further substantiates the complexity of NF-κB dysregulation in mesothelioma^69,70^. Importantly, our findings establish a correlation between NF-κB dysregulation and the overexpression of FABP5, shedding light on a potential molecular link between lipid metabolism and inflammation in mesothelioma.

The therapeutic implications of targeting FABP5 in mesothelioma are underscored by the observed effects of FABP5 silencing on cell dynamics, metabolism, and NF-κB activity (Figs. 4 and 5). The reduction in proliferation rates and the induction of apoptosis in mesothelioma cells upon FABP5 silencing highlight the potential of FABP5 as a promising therapeutic target. The metabolic shifts, including reduced mitochondrial activity and alterations in key metabolic ratios, further emphasize the multifaceted impact of FABP5 on cellular metabolism. Crucially, our study unveils a potential crosstalk between FABP5 and NF-κB, where FABP5 silencing leads to a modulation of NF-κB activity and downstream inflammatory gene expression. Regarding the possible molecular mechanism through which FABP5 could alter NF-κB activity, it is worth noting that among the interactors of FABP5 are the protein CYLD (Biogrid 107920) and the protein BAP1 (BioGRID:113911). CYLD is a well-known regulator of NF-κB pathway, through its de-ubiquitinase activity on IKK regulatory proteins, including TRAF2 and TRAF6^71–74^. BAP1 is a crucial tumor suppressor gene frequently mutated or inactivated in pleural mesothelioma (PM) and plays a pivotal role in regulating various cellular processes, including DNA damage repair, cell cycle regulation, and chromatin remodeling. Its loss or mutation is associated with increased genomic instability, which promotes tumorigenesis in mesothelial cells. In mesothelioma pathogenesis, BAP1 dysfunction contributes to altered cellular growth, enhanced survival, and resistance to apoptosis. The loss of BAP1 expression also impacts inflammatory pathways and immune evasion, further facilitating cancer progression. BAP1 has been linked to a specific mesothelioma subtype with distinct clinical and histopathological features, often associated with a relatively better prognosis compared to other PM subtypes. Given its key role in tumor suppression, BAP1 has emerged as a potential biomarker for diagnosis, prognosis, and therapeutic targeting in mesothelioma, making it an essential component in understanding the molecular landscape of PM. The consistency of the trends observed in our models (IST-Mes2 and MPP89 cell lines) suggests a broader applicability of our findings across different mesothelioma subtypes. While the study offers valuable insights into the connections between cellular metabolism, NF-κB dysregulation, and mesothelioma pathogenesis, it is important to acknowledge the limitations in clinical translation, and generalizability of findings. Addressing these limitations will be crucial for advancing our understanding of mesothelioma biology and developing effective therapeutic interventions. From a clinical perspective, these findings could inform the design of future diagnostic tools or biomarkers for mesothelioma, allowing for more precise stratification of patients based on metabolic profiles. Additionally, targeting FABP5 and its associated metabolic pathways may improve treatment outcomes for mesothelioma patients, particularly those resistant to current therapeutic approaches. Of course, clinical trials and preclinical studies in relevant animal models are necessary to validate the efficacy and safety of targeting FABP5 as a therapeutic strategy for PM. Our comprehensive investigation unravels the intricate connections between growth dynamics, lipid metabolism, and NF-κB dysregulation in mesothelioma. The distinctive metabolic signatures and the identified molecular players, particularly FABP5, present potential avenues for targeted therapeutic interventions. Overall, the study offers valuable insights into the molecular mechanisms underlying PM pathogenesis and potential therapeutic strategies.

## Materials and methods

### Cell lines, plasmids, siRNA, transfection, and luciferase assay

PM cell lines, MPP89 and IST-Mes2, as well as HMCs were cultured in DMEM F-12 medium supplemented with 10% FBS and maintained at 37 °C in a 5% CO2-humidified atmosphere. Cell culture media were supplemented with 10% fetal bovine serum (FBS), 2 mM L-glutamine, 1 mM Na-pyruvate, 50 mM 2β-mercaptoethanol, 100 U/mL penicillin, and 100 μg/mL streptomycin; all reagents were purchased from Thermo Fisher Scientific. pκBluc and pSV-β-Gal were purchased from Promega (Madison, WI, USA). HMC, MPP89, and IST-Mes2 were transfected with DNA by using Lipofectamine™ 2000 Transfection Reagent (Invitrogen, Carlsbad, CA, USA), according to the manufacturer’s protocol; total DNA amounts were equalized by transfection of pRc/CMV empty vector (Invitrogen, Carlsbad, CA, USA). For luciferase assays, pSV-β-Gal was co-transfected with pκBluc to monitor the transfection efficiency. Forty-eight-hour post-transfection, cells were lysed in lysis buffer of Dual Light Luciferase System (Tropix, Bedford, MA, USA) and the luciferase and β-galactosidase activities were evaluated by using Dual Light Luciferase System (Tropix) in a bioluminometer (Turner Biosystem, Sunnyvale, CA, USA). The ratio of firefly luciferase activity to β-galactosidase activity was expressed as relative light units^75,76^. RNA interference experiments for FABP5 silencing were performed by transfecting, through Lipofectamine 2000, ON-TARGETplus Smartpool Human FABP5, or Smartpool nontargeting (control) (Thermo Scientific Dharmacon).

### Cell proliferation, viability, apoptosis

For the monitoring of cell proliferation and viability, 1 × 10^6^ cells of PM cell lines, MPP89 and IST-Mes2, as well as HMC, were plated and maintained in culture for 24, 48, and 72 h. The number of viable cells was determined using trypan blue dye exclusion while the total cell viability was determined by CellTiter-Glo® Luminescent Cell Viability Assay (Promega, Madison, WI, USA). Cell cycle analysis was performed as previously described^77^. Briefly, cells were fixed with 70% (v/v) cold ethanol and stored at −20 °C for 1 h. Then, cells were washed with cold PBS, centrifuged and the pellets were suspended in 200 μL of non-lysis solution containing PI (50 μg/mL) and RNase (250 μg/mL). After incubation at 4 °C for 30 min, cells were analyzed by FACS BD LSRFortessaTM X-20 cytofluorometer (BD Biosciences) and data were processed with FlowJo software (Tree Star, Inc.). Annexin V-based apoptotic assay was performed as previously described^78,79^. Briefly, MPP89, IST-Mes2, and HMC (1 × 10^6^) were stained with FITC-conjugated Annexin V by using the Annexin V-FITC kit (Miltenyi Biotech). Data were collected by a FACS BD LSRFortessaTM X-20 cytofluorometer (BD Biosciences) and data were processed with FlowJo software (Tree Star, Inc.).

### Lipidomic analysis

For GC-MS analyses, 3 × 10^6^ cells were scraped in ice-cold water and centrifuged at 10,000 × *g* for 5 min at 4 °C. An aliquot of the sample was analyzed to measure total protein content. For intracellular quantitation of fatty acids. Membrane pellets were dissolved in 1 mL of ice-cold dichloromethane, and supernatants dried and resuspended in acetonitrile. For extracellular quantitation of fatty acids, conditioned media were vacuum dried to be then dissolved in 1 mL of ice-cold dichloromethane, and supernatants dried and resuspended in acetonitrile. The remaining sample was solubilized in pyridine (50 μL) and derivatized with 25 μL of N,O-Bis(trimethylsilyl(TMS)trifluoroacetamide) (BSTFA) with a reaction time of 90 min. One μL was injected, split ratio 1:10. GC-MS analyses were carried out on a Shimadzu GCMS 2010plus (Kyoto, Japan) with the following parameters. Injection temperature 280 °C, Ramp 0–1.00 min 100 °C, 1.00–6.00 min 100–320 °C, hold for 2.33 min. Column flow 1.10 mL/min Linear velocity 39 cm/s. Helium gas was used. Ion source Temp. 200 °C, Interface 320 °C, Solvent cut 5.9 min, Scan 35–600 m/z. Detector voltage 0.1 kV. Separation was performed on an Agilent (Santa Clara, CA, U.S.A.) SIL-8, 30 × 0.25 mm, 0.25 μm. Fatty acids were assigned by comparing their Retention index and fragmentation pattern to pure standards (Supplementary Fig. 1 and Supplementary Table 1). Quantitative measurements were achieved by building calibration curves (Supplementary Fig. 2 and Supplementary Table 2) of deuterated standards dissolved in cell extracts. Intracellular and extracellular content of fatty acid were expressed as pg/μg of total protein content. For normalization, metabolite content was obtained by calculating the pg of metabolites in a specific sample of the total protein content of the same sample (Supplementary Table 2). Comparisons and differences were analyzed for statistical significance by two-way ANOVA test and Bonferroni post-tests analysis. Representative fragmentation pattern of Stearic acid TMS (upper panel), Palmitic acid TMS (middle panel) and Myristic acid TMS (lower panel) have been shown in Supplementary Fig. 1. Overlayed chromatographic profiles of lipids extracted from HMC35 (green) cells or IST-MES-2 (red) and MPP89 (blue) mesothelioma cells and derivatized with TMS (Supplementary Fig. 2). The lower panels represent chromatographic regions corresponding to TMS palmitic acid (lower left) and TMS stearic acid (lower right) (Supplementary Fig. 2).

### Measurement of Intracellular ROS

For the monitoring of ROS production, 1 × 10^6^ cells were stained with CM-H2DCFDA 5 µM for 30 min (C6827, Thermo Fisher, Carlsbad, USA) according to the manufacturer’s instructions. CM-H2DCFDA fluorescence was analyzed by fluorimeter (Cary Eclipse fluorescence spectrophotometer (Varian)) for the measuring of Fluorescence Intensity (F.I.). Three independent experiments were performed. Data were further plotted and statistical analysis performed in GraphPad Prism9.

### Measurement of mitochondrial activity

To measure changes in the mitochondrial activity, 1 × 10^6^ HMC, IST-Mes2, or MPP89 cells, transfected with FABP5 siRNA, control siRNA, or left untransfected were reacted with Mitotracker Red CMXRos probe (MitoTracker™ Red CMXRos Dye, Thermo Fisher, Carlsbad, USA) 100 nM for 30 min at 37 °C, according to the manufacturer’s instructions. Then cells were washed three times with cold phosphate-buffered saline (PBS) 1X and then Mitotracker Red CMXRos fluorescence was analyzed by fluorimeter (Varian) for the measuring of Fluorescence Intensity (F.I.). Three independent experiments were performed. Data were further plotted and statistical analysis performed in GraphPad Prism9.

### ADP/ATP ratio measurement

To measure *ADP/ATP ratio*, 1 × 10^6^ HMC, IST-Mes2, or MPP89 cells, were transfected with FABP5 siRNA, control siRN,A, or left untransfected. Cells were washed twice with cold PBS 1x and the measurement was performed using the luminometric ATP/ADP ratio assay kit (from Sigma-Aldrich, St Louis, MO, USA, MAK135) following the manufacturer’s instruction. The luminescence was measured using luminometer (Turner BioSystems 20/20 Luminometer, Promega). Three independent experiments were performed. Data were further plotted and statistical analysis performed in GraphPad Prism9. The results were expressed as ratio relative levels of ADP on ATP.

### NAD +/NADH ratio measurement

To measure *NAD*+*/NADH ratio*, 1 × 10^6^ HMC, IST-Mes2, or MPP89 cells, were transfected with FABP5 siRNA, control siRNA, or left untransfected. The measurement was performed using the fluorimetric NAD+/NADH ratio assay kit (from Sigma-Aldrich, MAK460) following the manufacturer’s instruction. Briefly, cells were washed twice with cold PBS 1x. Extract cells were resuspended in 500 μl of NAD+/NADH Extraction Buffer by freeze/thawing for 2 cycles of 20 min on dry ice followed by 10 min at room temperature. After centrifugation, the surnatant was transferred into a labeled tube. The NAD+/NADH assay kit is based on a lactate dehydrogenase cycling reaction, in which the formed NADH reduces a probe into a high fluorescence product. The fluorescence intensity was measured at *λEx *= 530 nm/*λEm *= 585 nm, and was proportional to NAD+/NADH concentration in the sample. The fluorescence was measured using fluorimeter (Biotek Synergy H, Agilent Technology). Data were further plotted and statistical analysis performed in GraphPad Prism9. The results are expressed as ratio relative levels of NAD+ on NADH.

### Quantification, visualization, and analysis of lipid droplets

For the quantification of lipid droplets, 1 × 10^6^ HMC, IST-Mes2 or MPP89 cells were stained with BODIPY™ 493/503 (4,4-Difluoro-1,3,5,7,8-Pentamethyl-4-Bora-3a,4a-Diaza-s-Indacene, D3922, Thermo Fisher, Carlsbad, USA) 2.5 µM for 30 min. After the different staining, cells were washed two times with cold PBS and then analyzed by fluorimeter (Cary Eclipse fluorescence spectrophotometer (Varian)) for the measuring of Fluorescence Intensity (F.I.). Data were further plotted and statistical analysis performed in GraphPad Prism9. For lipid droplets visualization, 1 × 10^6^ HMC, IST-Mes2, or MPP89 cells were stained with BODIPY™ 493/503 2.5 µM for 30 min. Cells were grown in 6-well plates containing glass coverslips, in presence of full growth medium. After reaching 70–80% confluence, the cells were fixed in 4% paraformalehyde for 20 min at room temperature, permeabilized using 0.2% Triton-X 100 for 15 min at room temperature, and the lipid droplets (LDs) were stained with 5 μg/ml BODIPY 493/503 for 30 minutes at room temperature. The coverslipd were mounted on microscope slides using DAPI Fluoromount-G (SouthernBiotech, Cat.# 0100-20). Images were acquire using a Leica STELARIS 5 confocal Microscope using a 63× objective. At least 7 sites per sample were acquired.

### Cell extracts, western blotting, IKK activity, NF-κB DNA binding and TNF-α treatments

Nuclear and cytosolic extracts were performed as previously described^80^. Western blotting analysis was performed by resuspending protein aliquots in loading buffer (125 mM Tris–HCl, pH 6.8, 5% SDS, 1% bromophenol blue, 10% β-mercaptoethanol, 25% glycerol), resolved on 12% SDS–PAGE, transferred to nitrocellulose membrane (Millipore, Bedford, MA, USA) and incubated with primary antibodies (1:1000) followed by incubation with horseradish-peroxidase-linked mouse or rabbit IgG (1:2000)^81^. Primary antibodies were purchased from: Santa Cruz Biotechnology, Santa Cruz, CA, USA (anti-Histone H1, (H-2) sc-393358, anti-Vinculin, (7F9) sc-73614, anti-IKK alpha, (B-8) sc-7606); Upstate, Lake Placid, NY, USA (anti-p65), 06-418; Cell Signaling Technology, Danvers, MA, USA: Phospho-IKKα/β (Ser176/180) (16A6), mAb #2697, and anti-FABP5 (D1A7T) Rabbit mAb #39926. Secondary antibosies were purchased by Biorad, Goat Anti-Rabbit IgG (H + L)-HRP Conjugate, 1706515, and Goat Anti-Mouse IgG (H + L)-HRP Conjugate, 1706516. IKK activity was evaluated in cytosolic extracts using the HTScan IKK kinase assay kit (Cell Signaling Technology, Danvers, MA, USA). Binding of p65, p50, cRel and RelB to the double-stranded NF-κB oligonucleotide was measured using NF-κB Combo Transcription Factor Assay kit (Cayman Chemical Company, Ann Arbor, MI, USA)^82^. Cells were treated with TNF-α (20 ng/ml, Sigma-Aldrich, St Louis, MO, USA) for 45 min.

### Real-time PCR

Total RNA was extracted from cells by using the TRIzol reagent (Invitrogen); RNA aliquots (200 ng) were reverse transcribed using Random Examers (Roche) and Superscript III Reverse Transcriptase (Invitrogen), according to the manufacturer’s protocol. Real-time PCR was performed with the iQ Green Super mix (Bio-Rad Laboratories) and carried out with the iCycler iQ Real-Time detection system (Bio-Rad Laboratories) under the following conditions: 95 °C, 1 min; (94 °C, 10 s; 60 °C, 30 s) × 40. Primers are listed in Supplementary data. Real-time PCR of NF-κB-dependent genes was performed using the RT^2^ profiler PCR Array-Human NF-κB signaling pathway (QIAGEN Sciences, MD, USA). Reactions were carried out in triplicate, and gene expression levels were calculated relative to GAPDH mRNA levels as endogenous control. Relative expression was calculated as 2 ^(Ct gene under investigation − Ct GAPDH)^^83^.

### Chromatin immunoprecipitation assay

Cells were fixed by adding formaldehyde (Sigma-Aldrich) at the final concentration of 1%. After 10 min, ice-cold PBS plus 0.125 M glycine was added, and plates were transferred on ice, washed extensively with PBS, and scraped. After centrifugation, cells were 10 min lysed in lysis buffer (5 mM PIPES pH 8.0, 85 mM KCl, 0.5% NP-40) supplemented with 1× Complete Protease Inhibitor (Roche Diagnostic GmbH). Nuclei were pelletted (1000 × *g*, 5 min), and resuspended in sonication buffer (50 mM Tris–HCl pH 8.0, 1% SDS, 10 mM EDTA). Chromatin was sonicated using Bandelin Sonoplus GM70 (Bandelin Electronic, Berlin, Germany), centrifuged (14,000 × *g*, 15 min), and supernatant was 10-fold diluted in dilution buffer (0.01% SDS, 16.7 mM Tris–HCl pH 8.0, 1.1% Triton X-100, 167 mM NaCl, 1.2 mM EDTA). Samples were pre-cleared by 3-h incubation with 20 µl of protein G agarose beads followed by incubation with antibodies against the analyzed proteins. Primary antibodies were: anti-p65 (sc-372), and rabbit IgG (sc-2027) from Santa Cruz Biotechnology. Immunoprecipitations were carried out at 4 °C overnight and immune complexes were collected with protein G agarose beads, washed five times with low salt buffer (20 mM Tris–HCl pH 8.0, 0.1% SDS, 1% Triton X-100, 2 mM EDTA, 150 mM NaCl), four times with high salt buffer (20 mM Tris–HCl pH 8.0, 0.1% SDS, 1% Triton X-100, 2 mM EDTA, 500 mM NaCl), once with TE buffer (10 mM Tris–HCl pH 8.0, 1 mM EDTA), and extracted in TE buffer containing 2% SDS. Protein–DNA cross-links were reverted by heating at 65 °C overnight. DNA was further purified by QIAquick PCR purification kit (QIAGEN) and eluted in 50 µl sterile distilled water. Specific enrichment in NF-κB enhancer sequences was measured by real-time PCR of chromatin immunoprecipitation (ChIP) eluates using SYBR GreenER Master Mix (Invitrogen). Reactions were carried out with the iCycler iQ Real-Time detection system (Bio-Rad Laboratories) using the following conditions: 95 °C, 1 min; (94 °C, 10 s; 60 °C, 30 s) × 40. For each sample, values were normalized to input DNA and reported as % of input over the rabbit IgG control^84^. Specific primers for chromatin immunoprecipitation experiments are listed in Supplementary data.

### Statistics and reproducibility

Statistical analysis were performed using GraphPad Prism or Excel. Statistically differences were performed by using two-tail unpaired Student’s *t*-test. Data were reported as means ± SE. Differences between the means were considered as statistically significant at the 95% level (*p*  <  0.05). Comparisons and differences between metabolites signals were analyzed for statistical significance by two-way ANOVA test and Bonferroni post-tests analysis. In particular, two-way ANOVA was used to analyze multiple experimental conditions, Bonferroni post-test was used to determine the significance of each pair of conditions tested. The volcano plot related to the analysis of NF-κB-dependent genes array highlights upregulated or downregulated genes with the following parameters: log2 fold change (log2FC) > 1.5 or <−1.5; significance (−Log10 of *p* value (calculated by two-tail unpaired Student’s *t*-test) > 2).

### Reporting summary

Further information on research design is available in the Nature Portfolio Reporting Summary linked to this article.

## Supplementary information

Supplementary Information

Supplemental Data 1

Supplemental Data 2

Description of Additional Supplementary Materials

Reporting Summary

## Data Availability

The numerical source data for graphs in the main text are available in Supplementary Data 1. The uncropped blot images for figures in the main text are available in Supplementary data 2. The other data that support the findings of this study are available from the corresponding author upon reasonable request.
