# Supplementary material for: FABP5 is a key player in metabolic modulation and NF-κB dependent inflammation driving pleural mesothelioma
Source: Commun Biol. 2025 Feb 27;8:324. doi: 10.1038/s42003-025-07754-0 (PMC11868402; doi:10.1038/s42003-025-07754-0)
Supplement: Supplementary file 1 — Supplementary Information [file 42003_2025_7754_MOESM1_ESM.pdf]

Supplementary materials

FABP5 is a Key Player in Metabolic Modulation and NF-κB dependent Inflammation driving Malignant Pleural Mesothelioma

[1,*]Eleonora Vecchio, [1,*]Raffaella Gallo, [1,*]Selena Mimmi, [1]Debora Gentile, [1]Caterina Giordano, [1]Emilio Straface, [2]Rossana Marino, [2]Carmen Caiazza, [3]Arianna Pastore, [2]Maria Rosaria Ruocco, [4]Alessandro Arcucci, [5]Marco Schiavone, [1]Camillo Palmieri, [1]Enrico Iaccino, [3]Mariano Stornaiuolo, [1]Ileana Quinto, [2]Massimo Mallardo, [6]Fernanda Martini, [6,§] Mauro Tognon and [1,§,#]Giuseppe Fiume

[1] Department of Experimental and Clinical Medicine, University of Catanzaro "Magna Graecia", 88100 Catanzaro, Italy

[2] Department of Molecular Medicine and Medical Biotechnology, University of Naples "Federico II", Via S. Pansini 5, 80131 Naples, Italy

[3]Department of Pharmacy, University of Naples Federico II, Naples, Italy.

[4] Department of Public Health, University of Naples "Federico II", Via S. Pansini 5, 80131 Naples, Italy

[5]Department of Molecular and Translational Medicine, Zebrafish Facility, University of Brescia, 25123 Brescia, Italy.

[6] Department of Medical Sciences, University of Ferrara, Ferrara, Italy.

* These authors contributed equally to this paper

§ These authors should be considered as co-last authors

# Correspondence should be addressed to Prof. Giuseppe Fiume, fiume@unicz.it

Keywords: Pleural Mesothelioma, Cancer Metabolism, NF-κB, FABP5, Inflammation

Supplementary Material and Methods

Primers for Quantitative Real-Time PCR (RT-PCR)

Quantitative Real-Time PCR experiments were performed by using the following primers: SLC27A1 (Fw: GCAAGAAATTCTCGGCCAGC; Rev: TCCGTGAACTCCTCCCAGAT); SLC27A4 (Fw: GTTCCGCTGGAAAGGTGAGA; Rev: GCATACAGGGGCAGTTCCTT); SLC27A5 (Fw: AGCCCTGCCCTCTTCATCTA; Rev: CCCAACGACAAGTCCCATCA); FABP1 (Fw: ATCGTGCAGAATGGGAAGCA; Rev: CCGTTGAGTTCGGTCACAGA); FABP3 (Fw: CAGCATGACCAAGCCTACCA; Rev: CTCTTGCCCGTCCCATTTCT); FABP4 (Fw: GCTTTGCCACCAGGAAAGTG; Rev: TGCACATGTACCAGGACACC); FABP5 (Fw: CAGTTCAGCAGCTGGAAGGA; Rev: TGCCATCAGCTGTGGTTTCT); CD36 (Fw: TGCAGCCTCATTTCCACCTT; Rev: GGGTTTTCAACTGGAGAGGC); GAPDH (Fw: GTATGACTCCACTCACGGCAAA; Rev: TTCCCATTCTCGGCCTTG); XBP1s (Fw: AAGAACACGCTTGGGAATGG; Rev: CTGCACCTGCTGCGGAC); PCYT1A/CCTalpha (Fw: AACTCCTTGTGAGCGACCTG; Rev: TGCGTCATAGCGCTCATTCT); CHPT1 (Fw: TTGCGCTCATTGGCAGACTT; Rev: CATTCTTGCCAACACCACCA); CEPT1 (Fw: CCTACAGCTACAGAGCAGGC; Rev: CCAATCAGGGTTTGTCCCCA); CCL2 (Fw: GCAACCAGTTCTCTGCATCA; Rev: TGGCTGCTC GTCTCAAAGTA); IL1β (Fw: AGCTACGAATCTCCGACCAC; Rev: CGTTATCCCATGTGTCGAAGAA); IL6 (Fw: ACTCACCTCTTCAGAACGAATTG; CCATCTTTGGAAGGTTCAGGTTG); IL8 (Fw: GTTTTTGAAGAGGGCTGAGAATTC; Rev: ATGAAGTGTTGAAGTAGATTTGCTTG)

Primers for Chromatin Immunoprecipitation

Quantitative Real-Time PCR in Chromatin Immunoprecipitation experiments were performed by using the following primers: GAPDH (Fw: CCCATCACCATCTTCCAGGAG; Rev: GTTGTCATGGATGACCTTGGC); PCYT1A/CCTalpha kB promoter: (Fw: ACGTCTTCGCTGCATCCTCC; Rev: GGAAGAGCCAGCCGGAAGTTC); CHPT1 κB promoter (Fw: AATACATGGCAGGGCCGC; Rev: CCTGAGGTCTGATGTCTTAACCC); CEPT1

κB promoter (Fw: TGAGCCCAGCTGCACTCAT; Rev: GTCTGGTAAGGTAAGTCGAAGTAG)

Supplementary Tables and Figures

Supplementary Table 1

Metabolites	*m/z*	R_t	NIST Database Mass Spectrum
Myrstic acid, TMS (SFA C14:0)	285; 207; 132; 129; 117; 75; 55	16.7	https://webbook.nist.gov/cgi/cbook.cgi?ID=C112618&Mask=200#Mass-Spec
Palmitic acid, TMS (SFA C16:0)	313; 145; 132, 131, 129; 117; 75; 55	18.7	https://webbook.nist.gov/cgi/cbook.cgi?ID=C55520893&Mask=3FB7
Stearic acid, TMS (SFA C18:0)	341,145, 132, 129, 117, 75, 55	20.6	https://webbook.nist.gov/cgi/cbook.cgi?ID=C18748919&Mask=200

Supplementary Table 1. Metabolites attributed by GC-MS, their fragmentation pattern (MS/MS spectra), retention time (min) and link to the NIST database.

Supplementary Table 2

Compound	Linearity range (pg/ μg)	Calibration curve	R^2	LOD (pg/ μg)[1]	LOQ (pg/ μg)[1]
**Myrstic acid,TMS**	1 – 100	y = 341258x + 12527	0.99	0.06 ± 0.01	0.1 ± 0.05
**Palmitic acid, TMS**	1 – 100	y = 2377425x +40438	0.99	0.009 ± 0.003	0.05 ± 0.01
**Stearic acid, TMS**	1 – 100	y = 1065243x + 61643	0.99	0.007 ± 0.002	0.06 ± 0.01

Supplementary Table 2. Linearity range, equation of calibration curves, linearity, limit of detection (LOD) and limit of quantification (LOQ) values of the GC/MS analysis for the indicated derivatized fatty acids.

Supplementary Figure 1

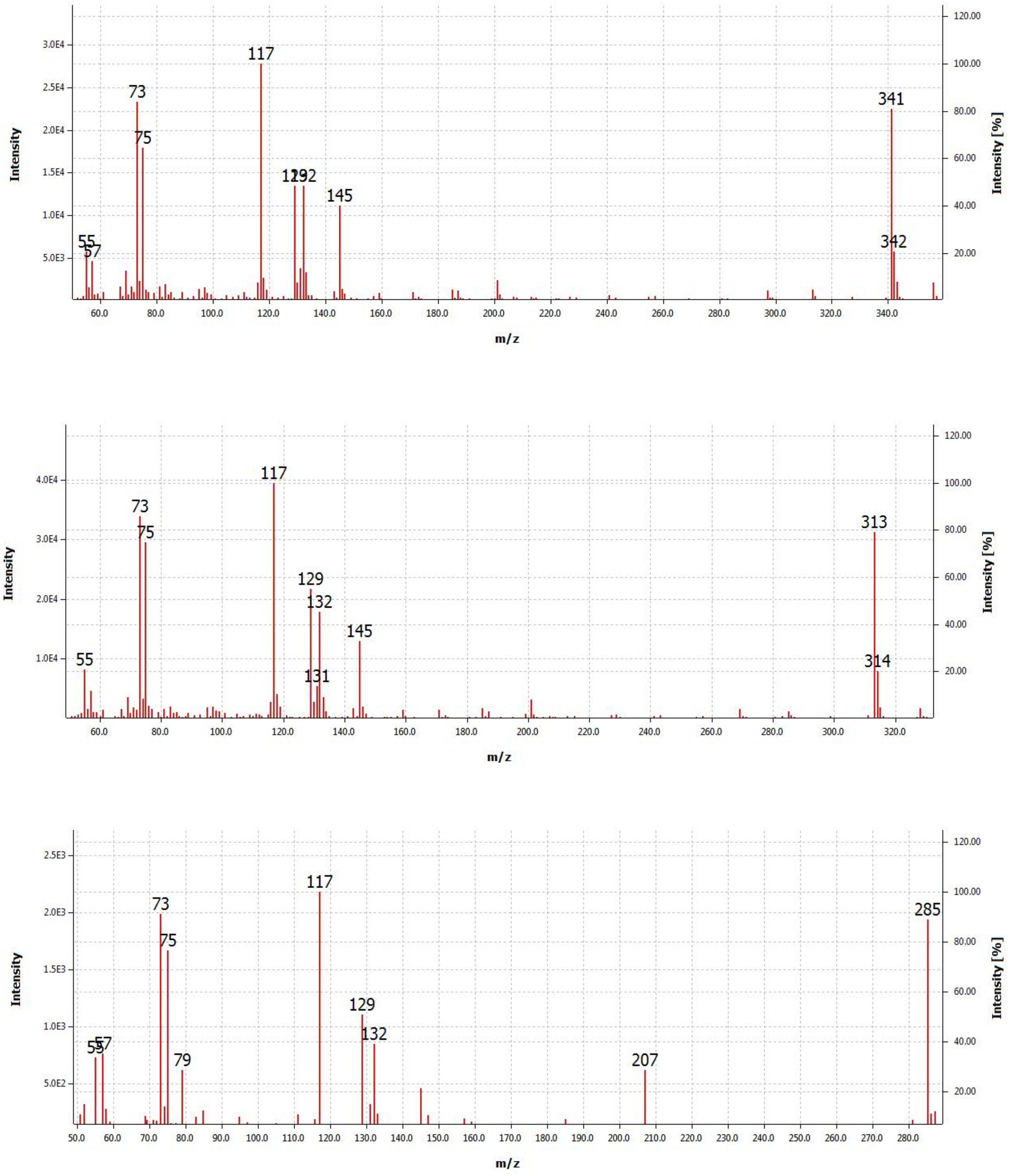

Supplementary Figure 1. Representative fragmentation pattern of Stearic acid TMS (upper panel); Palmitic acid TMS (middle panel) and Myristic acid TMS (lower panel) in samples from HMC35, IST-MES-2 or MPP89 mesothelioma cells.

Supplementary Figure 2

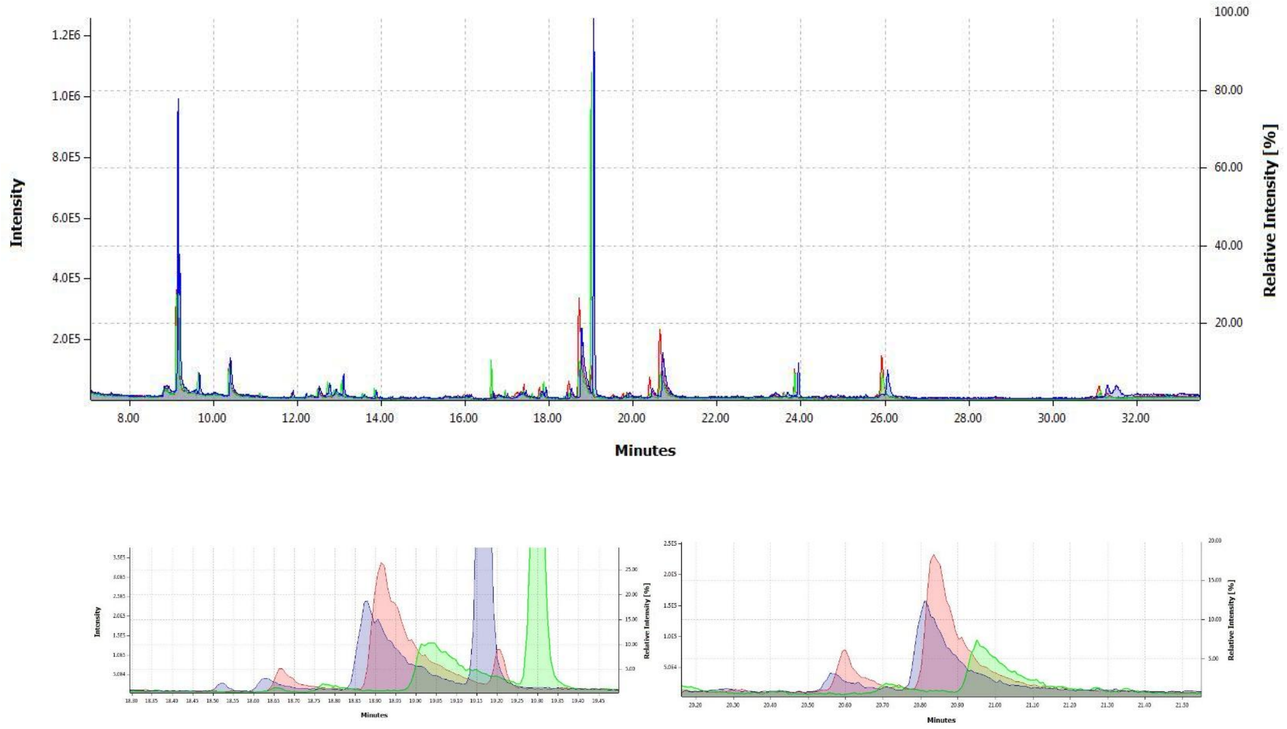

Supplementary Figure 2. Upper panel: overlayed chromatographic profiles of lipids extracted from HMC35 (green) cells or IST-MES-2 (red) and MPP89 (blue) mesothelioma cells and derivatized with TMS as described in the method section. The overlay is representative of three independent experiments. The lower panels represent chromatographic regions corresponding to TMS palmitic acid (lower left) and TMS stearic acid (lower right). Chromatograms were manually shifted from each other of 0.1 minutes for clarity.

Supplementary Figure 3

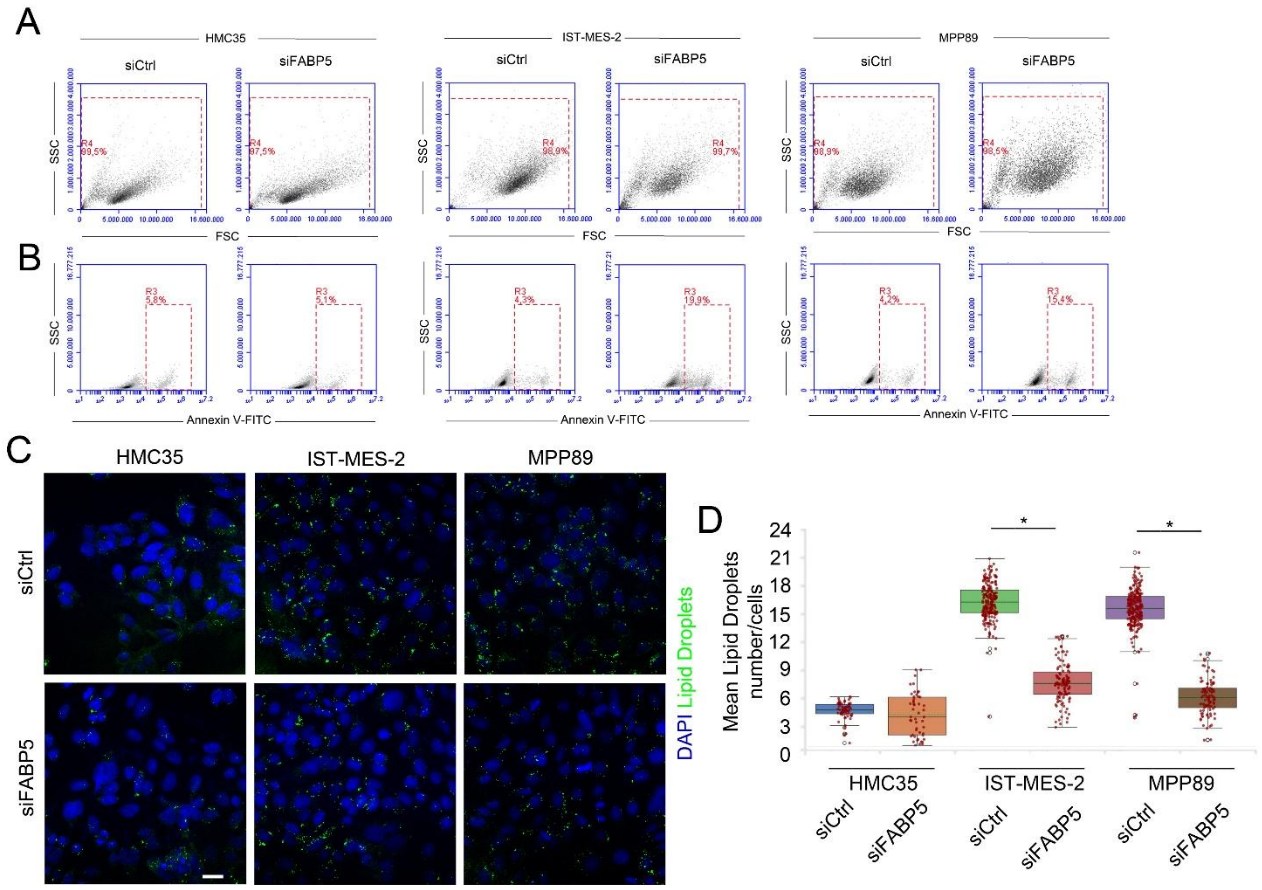

Supplementary Figure 3. (A) Gating strategy of IST-Mes2 and MPP89, and HMC35 cells transfected with siRNA FABP5 or scrambled siRNA via flow cytometry. Cells were gated from FSC and SSC based on size and granularity. **(B)** Representative density plot of Annexin V binding assay of IST-Mes2 and MPP89, and HMC35 cells transfected with siRNA FABP5 or scrambled siRNA analyzed by flow cytometry. **(C)** Confocal Fluorescence Images showing a higher number of Lipid Droplets per cell in IST-Mes2 and MPP89, and HMC35 cells transfected with siRNA FABP5 or scrambled siRNA. Images are representative of at least two experiments. Scale bar = 10 μm. **(D)** A boxplot reporting the Mean Lipid Droplet number per cell ± standard deviation is shown.
