# Supplementary material for: FABP5 is a key player in metabolic modulation and NF-κB dependent inflammation driving pleural mesothelioma
Source: Commun Biol. 2025 Feb 27;8:324. doi: 10.1038/s42003-025-07754-0 (PMC11868402; doi:10.1038/s42003-025-07754-0)
Supplement: Supplementary file 3 — Supplemental Data 2 [file 42003_2025_7754_MOESM3_ESM.pdf]

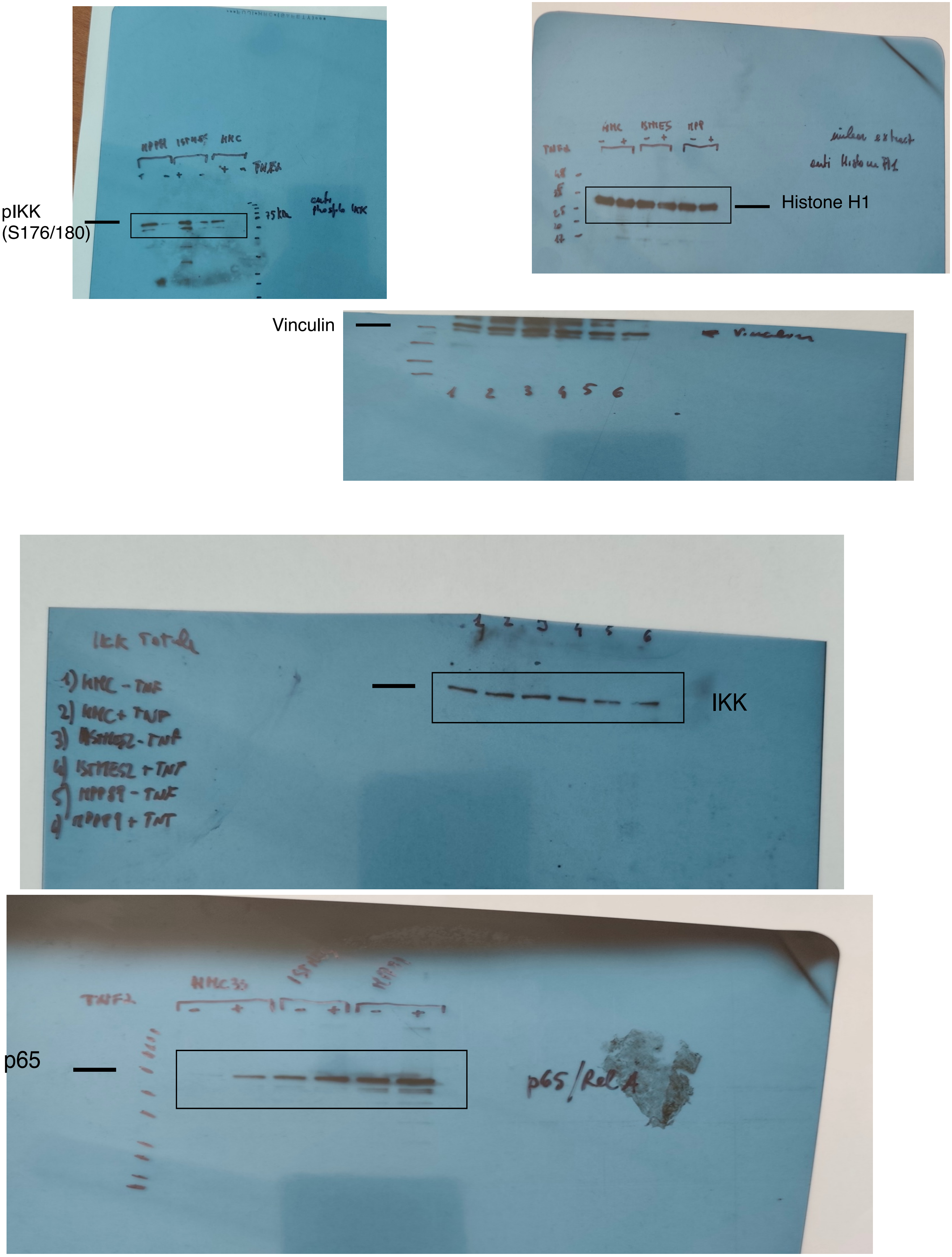

Supplementary Data 2. Presentation of original immunoblot shown in Figure 2C. The cropped parts of immunoblot were indicated with black boxes.

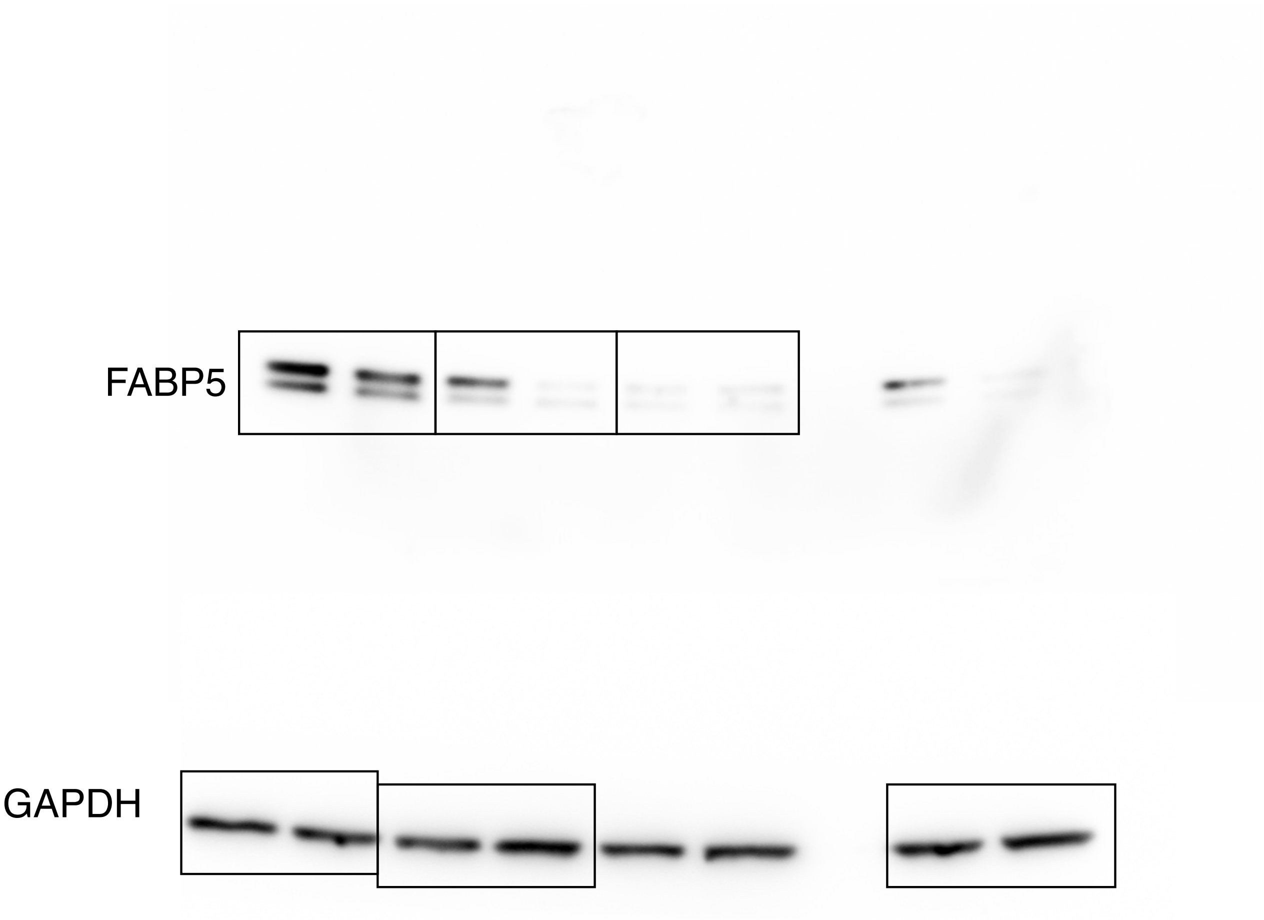

Supplementary Data 2. Presentation of original immunoblot shown in Figure 4B. The cropped parts of immunoblot were indicated with black boxes.
