# Supplementary material for: FABP5 is a key player in metabolic modulation and NF-κB dependent inflammation driving pleural mesothelioma
Source: Commun Biol. 2025 Feb 27;8:324. doi: 10.1038/s42003-025-07754-0 (PMC11868402; doi:10.1038/s42003-025-07754-0)
Supplement: Supplementary file 4 — Description of Additional Supplementary Materials [file 42003_2025_7754_MOESM4_ESM.pdf]

Description of Additional Supplementary Files

File name: Supplementary Data 1

Description: all the source data for graphs and charts

File name: Supplementary Data 2

Description: uncropped western blotting.
